# Laparoscopic Intracapsular Myomectomy in Women 40 Years Old and Over with Symptomatic Uterine Fibroids. A Pilot Study

**DOI:** 10.1055/s-0040-1722657

**Published:** 2021-03-22

**Authors:** Andrea Tinelli, Ioannis P. Kosmas, William H. Catherino, Jose Carugno, Ospan A. Mynbaev, Radmila Sparic, Giuseppe Trojano, Antonio Malvasi

**Affiliations:** 1 Department of Obstetrics and Gynecology, “Veris delli Ponti” Hospital, Scorrano, Lecce, Italy; 2 Department of Obstetrics and Gynecology, Ioannina State General Hospital G. Hatzikosta, University of Ioannina, Ioannina, Greece; 3 Department of Obstetrics and Gynecology, Uniformed Services, University of the Health Sciences, Bethesda, Maryland; 4 Division of Minimally Invasive Gynecology, Department of Obstetrics, Gynecology and Reproductive Science, University of Miami, Miller School of Medicine, Miami, Florida; 5 Laboratory of Human Physiology, Phystech BioMed School, Faculty of Biological & Medical Physics, Moscow Institute of Physics and Technology (State University), Dolgoprudny, Moscow Region, Russia; 6 Clinic for Gynecology and Obstetrics, Clinical Centre of Serbia, School of Medicine, University of Belgrade, Serbia; 7 Department of Obstetrics & Gynecology, Madonna delle Grazie Hospital, Matera, Italy; 8 Department of Obstetrics & Gynecology, Santa Maria Hospital, GVM Care & Research, Bari, Italy

**Keywords:** laparoscopic myomectomy, myoma pseudocapsule, reproductive outcome, menopause, pregnancy, uterine ageing

## Abstract

Authors evaluated the impact of laparoscopic intracapsular myomectomy (LIM) in women 40 years of age and over with desire of future fertility compared with medical management of symptomatic fibroids, by a prospective cohort study in University affiliated Hospitals. This study includes a cohort of women 40 years of age and older with symptomatic intramural fibroids with desire of future fertility. Women with symptomatic fibroid uterus were offered to undergo LIM or medical management. They were encouraged to attempt conception either spontaneously or by assisted reproductive technology (ART) according to their individual preference. All women were followed for 2 years. Fibroid characteristics, pre- and post-surgical variables, including surgical complications, days of hospitalization, pregnancy rate, and obstetrical outcomes were collected. A total of 100 patient were included in the analysis. Fifty patients were assigned to the LIM group and 50 to the medical treatment group (MT). Groups were similar regarding age (43.5 ± 2.4 and 43.5 ± 2.4, *p* = 0.99), body mass index (23.8 ± 3.1 and 24.2 ± 3.1, *p* = 0.54), parity (0.46 ± 0.09 and 0.58 ± 0.09, *p* = 0.37), fibroid number (1.38 ± 0.6 and 1.46 ± 0.6, *p* = 0.53), and fibroid size (5.92 ± 1.62 cm vs. 5.94 ± 1.49 cm, *p* = 0.949). Of the patients who underwent LIM, 62% conceived within the study period compared with 56% in the control group ( *p* = 0.54). Pregnancy was achieved by ART in 44% of the patients of the LIM group and 30% in control group. There was no significant difference in pregnancy rates among the two groups regarding spontaneous pregnancy rate ( *p* = 0.332), nor in pregnancies obtained by ART with own eggs ( *p* = 0.146) and oocyte or embryo donation ( *p* = 0.821). The take home baby rate was 65% (20/31) in the LIM group and 61% (17/28) in the control group ( *p* = 0.7851). Both groups had similar rate of miscarriage ( *p* = 0.748).

Patients 40 years old and over with symptomatic fibroid uterus who undergo LIM have similar subsequent fertility and obstetrical outcomes than women treated with medical management. LIM has no detrimental impact on future fertility in women 40 years old and over.

Uterine fibroids or leiomyomas are common benign tumors that cause anatomical disruption of the uterine architecture, leading to symptoms such as heavy or prolonged menstrual bleeding, pelvic pain, infertility, and recurrent pregnancy loss. Their effect on fertility depends on the location, with intramural and submucosal fibroids having the most significant negative impact. 1 2

Previous studies suggested that patients with fibroids distorting the uterine cavity had lower implantation rates and increased incidence of early pregnancy loss, when compared with women without fibroids. 1 2 Furthermore, in women with intramural fibroids, there is significantly lower rates of implantation, ongoing pregnancies, live births, and increased rates of spontaneous abortions. 3 Conversely, other studies report no adverse impact of fibroids on reproduction, implantation, and clinical pregnancy rates. 4 Moreover, a study published by Klatsky et al comparing fertility outcomes of women with or without uterine fibroids, focusing on pregnancy rates, concluded that no difference was found in the implantation or clinical pregnancy rates between the two groups. 5

The American Society of Reproductive Medicine (ASRM) stated that there is insufficient evidence to determine that a specific myoma size, number, or location (excluding submucosal myomas or intramural myomas impacting the endometrial cavity) is associated with adverse fertility outcomes in asymptomatic patients, acknowledging that there is insufficient evidence indicating that myomectomy in asymptomatic women, reduces pregnancy loss rates, unless the fibroid is distorting the uterine cavity. 6

The number of women who are intentionally delaying pregnancy has increased over the last 2 decades. 7 Fibroid uterus is a prevalent condition in woman of over the age of 40. Only two studies investigating the impact of myomectomy on fertility 8 9 have included patients 40 years old and older, and these studies did not stratify patients by age. 10 11

Since the debate about the clinical effect of nonendometrial cavity distorting intramural fibroids is ongoing and the number of patients 40 years old and over with symptomatic fibroids desiring future fertility is increasing, we aim to investigate the impact on clinical and fertility outcomes of laparoscopic intracapsular myomectomy (LIM) in patients of advanced reproductive age.

## Materials and Methods

**Fig. 1:**
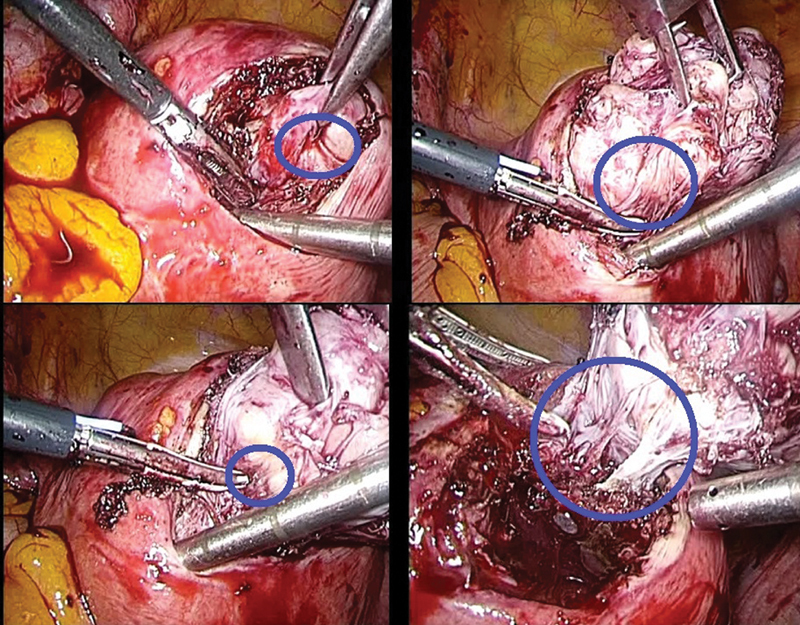
The myoma pseudo-capsule and myoma are highlighted in the blue circle: the uterine serosa was incised, and fibroid cleavage plane of the pseudo-capsule was entered (to enucleate the fibroid), preserving the myometrium below the pseudo-capsule, illustrated in clockwise sequence starting at the top left.

Women 40 years of age and older with symptomatic fibroids with desire of future fertility who were evaluated at the gynecology division of the “Veris delli Ponti” University Hospital between January 2013 and December 2017 were invited to participate. Patient were eligible to participate if meeting the following inclusion criteria: (1) age 40 year or over; (2) presence of up to three intramural fibroids (type 2–5 as per FIGO classification); (3) reporting having symptoms such as heavy menstrual bleeding, pelvic pain or pressure, frequent urination, constipation, or back pain (4) Desire of future fertility. Patients were excluded if they had: (1) submucous fibroids (Type 0 or 1 as per FIGO classification); (2) more than three fibroids; (3) suspected adenomyosis. Moreover, all patients with fertility-impairing metabolic diseases (as diabetes, thyroid disfunction, dysmetabolic diseases, etc.) were excluded from enrollment in the study. After informed consent was obtained, patient's demographic data was documented. A transvaginal pelvic ultrasound was performed to determine the number, size, and location of the fibroids. Patients were then offered one of two therapeutic options: LIM or MT with Ibuprofen, tranexamic acid, and oral progesterone. All the laparoscopic procedures (LIM) were performed by two experienced laparoscopists (A.T. and A.M.) following a previously published technique. 12 The fibroids cleavage planes were identified, and the fibroids were enucleated from their pseudo capsule with as minimal myometrial trauma as possible ( Fig. 1 ). Chromopertubation was performed to evaluate tubal patency and to facilitate the recognition of an inadvertently entered uterine cavity. The myometrium was closed in layer with 1/0 Vicryl (Ethicon, United States), including the overlying serosa, using a CT-1 curved needle, with intra or extracorporeal knots ties, or by unidirectional running barbed sutures by V-Lock (Medtronic, MN). The edges of the serosal uterine defect were approximated with introflexion U-inverted stitches (myometrium/serosa-serosa/myometrium direction) at 1 cm increments (baseball-type stitch closure).

Patients were re-evaluated at a follow-up visit 30 days after the surgery and had transvaginal ultrasound (US) at 60 and 90 days after surgery. The myometrial healing pattern was compared with the previous myometrial area occupied by the fibroids. After 90 days the uterine scars were considered healed and patients were encouraged to attempt conception either spontaneously or by assisted reproductive technology (ART), according to their preference. Fibroid characteristics, pre- and post-surgical parameters, including surgical complications and days of hospitalization were collected. Participants were followed with phone interviews each 6 months for 2 years from the time of inclusion in the study. Pregnancy rate and obstetrical outcomes during the follow-up period were recorded and analyzed for all women. Patients who conceived during the study period were followed up until the resolution of the pregnancy.

### Statistical Analysis

Student *t* -test was used to compare continuous variables between the two groups. Chi-square test (likelihood ratio) has been used for bi-modal parameters. Summary statistics on all patients, are presented as frequencies for categorical variables and mean ± standard deviation for continuous variables. Statistical analysis was performed using JMP 9 and SPSS 21 software. A *p* -value <0.05 was considered statistically significant.

## Results

A total of 116 were invited to participate, 100 completed the study after 2 years of follow-up. Fourteen cases were lost to follow-up, while two other patients erased because the majority of data was missing. The 100 patients were equally distributed in the two groups, with mean age of 43.56 ± 2.45 for the LIM group and 43.58 ± 2.46 for MT group. No significant difference was observed between the two groups regarding age ( *p* = 0.998), body mass index BMI ( *p* = 0.545), and parity ( *p* = 0.376) ( Table 1 ). The mean number of fibroids in the laparoscopy group was 1.38 ± 0.6 while in the control group was 1.46 ± 0.67 ( *p* = 0.53). Only three patients (5.8%) had three fibroids in the laparoscopy group and five (9.8%) in the control group. There was no significant difference in fibroid size in patients who had only one fibroid ( *p* = 0.94), two fibroids ( *p* = 0.67), or three fibroids ( *p* = 0.673). There was no significant difference in the uterine zone (fundal, body, and cervix), where fibroids were located between the two groups ( Table 2 ).

**Table 1:** Demographic characteristics

	Laparoscopy *N* = 50	No laparoscopy *N* = 50	*p*
Age	43.48 ± 2.28	43.6 ± 2.42	0.799 b
BMI	23.82 ± 3.13	24.2 ± 3.12	0.545 b
Parity (children)	0.46 ± 0.06	0.58 ± 0.07	0.378 b
Parity ( *n* = 0)	31 (62%)	27 (54%)	0.669 a
Parity ( *n* = 1)	15 (30%)	17 (34%)	
Parity ( *n* = 2)	4 (8%)	6 (12%)	

Abbreviation: BMI, body mass index.

a Chi-square test (likelihood ratio).

b *t* -Test.

**Table 2:** Fibroid characteristics

	Laparoscopy *N* = 50	No laparoscopy *N* = 50	*p*
Singular or multiple myomas	1.38 ± 0.6	1.46 ± 0.67	0.533 b
Number of fibroids/groups			
1	34	32	* 0.533 a*
2	13	13	
3	3	5	
Myoma size (cm) in patients with one fibroid	5.92 ± 1.62	5.94 ± 1.49	0.949 b
Fibroids at uterine zone 1 (fundal)	1.7 ± 0.7 ( *n* = 50)	1.68 ± 0.68 ( *n* = 50)	0.88 b
Fibroids at uterine zone 2 (body)	2.12 ± 0.15 ( *n* = 16)	2.22 ± 0.14 ( *n* = 18)	0.658 b
Fibroids at uterine zone 3 (cervix)	2 ( *n* = 3)	2.2 ± 0.83 ( *n* = 5)	0.7 b

a Chi-square test (likelihood ratio).

b *t* -Test.

In the LIM group, the mean duration of surgery was 96 ± 12,67 minutes. Preoperative mean hemoglobin (Hb) level was at 12.7 ± 1.07 g/dL and the mean postoperative Hb was 11.94 ± 1.12 g/dL. The length of stay was on average of 2 ± 0.4 days. About post-surgical complications, eight patients (16%) developed postoperative anemia and five (10%) developed postoperative fever (defined as a temperature greater than 38°C on 2 consecutive postoperative days) and treated with oral antibiotic.

Regarding fertility, 31 (62%) of the patients in the laparoscopy group and 28 (56%) in the control group conceived during the study period ( Table 3 ). Pregnancy was obtained by ART in 22 patients (44%) in the laparoscopy group and 15 (30%) in control group with no significant differences between the two groups in terms of overall pregnancy rate (0.541), spontaneous pregnancy (0.332), pregnancy achieved with ART with own eggs (0.146), and with oocyte or embryo donation (0.821). Regarding obstetrical outcomes, there was no significant difference in the patients who had spontaneous abortion in each group: 11 in the LIM group and 11 in the control group. In addition, there was no significant difference ( *p* = 0.748) between the two groups in terms of the gestational age at the time of the spontaneous abortion (10.36 ± 2.01 in the control and 10.09 ± 1.92 in LIM group). The mean time from surgery to conception in the LIM group was 13 months for the LIM and 16 months for the control group.

**Table 3:** Pregnancy rate and obstetrical outcomes

	LIM *N* = 50	Control *N* = 50	*p*
Overall pregnancies achieved	31 (62%)	28 (56%)	0.541 a
Spontaneous pregnancies	9 (18%)	13 (26%)	0.332 a
Overall, ART pregnancies	22 (44%)	15 (30%)	0.146 a
Oocyte donation pregnancies	13 (59%)	14 (93.33%)	0.821 a
Abortion (weeks)	10.09 ± 1.92 ( *n* = 11)	10.36 ± 2.01 ( *n* = 11)	0.748 b
Delivery (weeks)	37.2 ± 2.04 ( *n* = 20)	38.94 ± 1.14 ( *n* = 17)	0.0036 b
*Mode of delivery*	*LIM (n = 31)*	*Control (n = 28)*	0.163 a
CS	17	10	
Normal delivery	3	7	

Abbreviations: ART, assisted reproductive technology; CS, cesarean section; LIM, laparoscopic intracapsular myomectomy.

a Chi-square test (likelihood ratio).

b *t* –Test.

There was a significant difference between the two groups for gestational age of delivery ( *p* = 0.0036), favoring the control group. The laparoscopy group delivered at a mean gestational age of 37.2 ± 2.04 weeks and the control group at a mean gestational age of 38.94 ± 1.14 weeks, but none of the newborns needed assistance in the neonatal intensive care unit. Seventeen patients of the LIM group were delivered by cesarean section (CS) and 10 in the control group ( *p* = 0.07). All women gave birth to live fetuses, no obstetrical complications were reported.

Fibroids recurrence was not recorded during the 2 years of follow-up, the pathological examination revealed benign histology for all fibroids, no patients received blood transfusion during or after myomectomy.

## Discussion

The current pilot study highlights that LIM in women over the age of 40 with symptomatic fibroids has no detrimental effect on future fertility. More pregnancies were achieved in the LIM group than in the MT group, although without statistical significance, and the reproductive outcome was similar in both groups. In the last century, myomectomy for patients of advanced reproductive age was considered a rather controversial approach. Since the incidence of uterine fibroids increases with age and more women choose to delay childbearing, the prevalence of uterine fibroids during pregnancy is likely to increase. 13 Women, also those in the peri- or postmenopausal period, are less likely to accept the trauma of a hysterectomy for the management of a benign condition such as a symptomatic fibroid. 14 For patients with symptomatic fibroids or a significantly enlarged uterus, myomectomy should be a reasonable surgical option, especially for women with desire of future fertility. 15

Nevertheless, many gynecologists suggest patients to avoid myomectomy after the age of 40, because of the consequences of uterine aging, unknown postsurgical myometrial healing, and fear of leiomyosarcoma in women approaching menopause. 16 They are also reluctant due to the possible complications in pregnancy after myomectomy, such as the risk of abortion or uterine rupture. A recent study of laparoscopic myomectomy in patients during perimenopause confirmed the efficacy of this treatment, with low rate of complications and relapses as well as a high degree of patient satisfaction. 17

Our study revealed no difference in future fertility in patients undergoing myomectomy or those who avoided surgery, in terms of fertility rate: 31 (62%) pregnancies achieved in the laparoscopy group and 28 (56%) in the control group. A major disadvantage of myomectomy is a high recurrence rate, ranging from 9 to 50% or more at 5 years. However, there should be a low risk of reoperation in over 40 years age group of women. 18

Another concern of patients over the age of 40 years with intramural fibromas who desire to conceive, is the problems that fibroids can create during pregnancy. Uterine fibroids can be identified in approximately 8 to 20% of pregnant patients, during the first trimester US scan. Most of these patients can expect to have a normal pregnancy and delivery. Nevertheless, complications related to the presence of fibroids during pregnancy may occur depending on the size, number, and location of the fibroids. 19 Generally, during delivery, fibroids may rarely obstruct the normal labor course, and when CS is needed, can increase the complexity and risks associated with of the procedure. Uterine fibroids can increase operative delivery rate, post-partum bleeding, and infections, sometimes requiring transfusion for post-partum hemorrhages and occasionally leading to life-threatening postpartum hemorrhage. 20 21 The topic is debated, since there are studies reporting no adverse obstetric outcome in pregnant women with fibroids. 13 22 Fibroids also have been linked to increased risk of miscarriage by approximately 60%, with some estimates of risk as much as threefold greater. 23 Conversely, the presence of fibroids was not associated with increased risk of spontaneous abortion in an analysis of more than 20,000 pregnant women. 24 The variation in the literature likely reflects the difficulty in defining which women will be at risk of adverse obstetric outcome based on the number, size, and location of her fibroids.

Even if the influence of pregnancy on uterine fibroid size still remains an unsolved dilemma, the preventive removal of fibroids could reduce these much-discussed issues, especially in women over the age of 40 years in whom the uterine muscle fibers are more rigid, due to the effect of aging, which is associated with infertility and adverse pregnancy outcomes. 25 Moreover, there are significant ethical considerations and maternal and fetal complications related to pregnancy in women of advanced age, this risk can be mitigated by ensuring optimal preconceptional health status. 26 Some women of advanced age who, in the past, underwent ART with their own oocytes are now able to undergo a heterologous fertilization with oocytes of younger donors. Wang et al 23 recently affirmed that no cavity-distorting intramural fibroids would significantly reduce implantation rate, clinical pregnancy rate, and live birth rate and significantly increase the miscarriage rate after IVF treatment, but it would not significantly increase the ectopic pregnancy rate.

Kameda et al 24 reported on pregnancy rates in patients with infertility of unknown etiology other than the presence of uterine fibroids, concluding that laparoscopic myomectomy should be recommended in patients with fibroids and no obvious cause for infertility. Our data would reassure to proceed with myomectomy in patients over the age of 40 years with fibroid uterus and support the finding of a reduced myometrial healing time after myomectomy. Tsuji et al performed magnetic resonance imaging studies to evaluate the myometrial healing after myomectomy concluding that the recovery process is complete at 12 weeks after the operation. 25 Subsequently, it is advisable to recommend starting to spontaneously conceive or with a program of ART thereby decreasing the time from surgery to implantation to conception. The use of ARTs, such as oocyte donation, is a valuable therapy for women of advanced maternal age and in such cases, the woman can carry a pregnancy with an almost similar risk profile to that of her younger counterparts. 26

The impact of myomectomy in symptomatic women over the age of 40 years who desire to conceive is poorly investigated in literature. Obed et al 27 performed 98 abdominal myomectomies in women aged 40 years and above, the mean age of the patients was 42.6 ± 2.9 years and 77 (78.6%) of them were nulliparous. Full-term pregnancies were achieved in 72.7% of patients. Roux et al 28 in a retrospective study of a case series, analyzed the reproductive outcome after abdominal myomectomy among 34 patients older than 38 years. Three patients spontaneously conceived and two (13%) had an uncomplicated full-term delivery.

This study presents with strengths and limitations. One of the strengths, is the age of evaluated women. Also, the surgery was performed only by the two surgeons and the patients were followed up for 2 years, or until the resolution of the pregnancy if they conceived during the follow-up period. We acknowledge some limitations of this study like the lack of randomization, as the patients independently decided to undergo surgery or not, those undergoing ART, it was performed in different centers using different protocols, as multicentric study.

## Conclusion

The study has shown that LIM has no adverse impact on future fertility in patients over the age of 40 with symptomatic fibroids compared with those receiving MT. LIM is a safe and feasible uterine sparing option for patients of advanced reproductive age wishing to preserve their fertility. Future randomized studies with a rigid protocol should be performed to confirm our findings.
